# Synergistic Mechanical and Tribological Optimization of High-Entropy (TiVCrMoW)B Ceramics

**DOI:** 10.3390/ma19214607

**Published:** 2026-10-30

**Authors:** Pengbo Zhao, Hao Li, Wei Li, Jiabin Wang, Tianen Yu, Chen Xu, Jinlong Chen, Zhaowu Wang, Jiawei Wei, Zhong Wan, Binbin Dong, Zhiyu Min, Hailong Wang

**Affiliations:** 1 School of Materials Science and Engineering, Luoyang Institute of Science and Technology, Luoyang 471023, China; 18003820787@163.com (J.W.); ywte2006@outlook.com (T.Y.); jlchen@lit.edu.cn (J.C.); zw_wang@lit.edu.cn (Z.W.); weijiaweicera@126.com (J.W.); wanzhong2020@yeah.net (Z.W.); dongbb@lit.edu.cn (B.D.); mindayu@163.com (Z.M.); 2 Henan Key Laboratory of Green Building Materials Manufacturing and Intelligent Equipment, Luoyang 471023, China; 3 School of Materials Science and Engineering, Zhengzhou University, Zhengzhou 450001, China; liw@zzu.edu.cn (W.L.); 119whl@zzu.edu.cn (H.W.); 4 College of Materials and Chemistry, China Jiliang University, Hangzhou 310018, China

**Keywords:** high-entropy ceramics, monoborides, spark plasma sintering, element segregation, isostructural, wear resistance

## Abstract

To address the inherent brittleness and insufficient wear resistance of conventional transition metal monoboride ceramics, as well as the application limitations caused by large interfacial mismatches triggered by adding heterogeneous secondary phases, we prepared (TiVCrMoW)B homology and isostructural high-entropy monoboride ceramics containing compositionally enriched isostructural microregions using spark plasma sintering. This ceramic inherits the orthorhombic Cmcm-type monoboride crystal structure. XRD analysis indicates a single phase within its detection limit, while EDS characterization reveals significant elemental segregation, forming in situ compositionally enriched isostructural microregions that are Ti-, Mo-, and W-rich. The interfaces between these regions are well bonded, and the relative density reaches 94.13%. Relying on this intrinsic microstructural architecture, the material is observed to concurrently improve hardness and toughness under the present testing conditions, achieving a Vickers hardness of 39.95 ± 4.32 GPa under a 0.5 N load and a fracture toughness of 3.39 ± 0.36 MPa·m^1/2^. Under 10 N reciprocating dry friction conditions, it exhibits stable friction behavior, with a steady-state coefficient of friction maintained at 0.52 and a volume wear rate as low as (1.11 ± 0.4) ×10^−6^ mm^3^/(N·m). The wear mechanisms are primarily early-stage abrasive wear, microcrack propagation, and localized layered spalling, with no adhesive wear or brittle fracture observed. The low wear rate arises from the combined effects of lattice distortion induced by high entropy, isostructural compositional-microregion toughening caused by elemental segregation, and fine-grain-induced retardation of microcrack propagation. This work confirms that a homology isostructural compositional-microregion structure architecture can contribute to the concurrent enhancement of hardness, toughness, and wear resistance of high-entropy monoboride ceramics, and provides a promising design strategy for wear-resistant ceramics without external secondary phases based on the high-entropy strategy.

## 1. Introduction

Wear can be defined as the progressive loss of material from a surface caused by mechanical action. Abrasive wear and erosive wear are two major types of wear: abrasive wear is caused by hard particles or asperities sliding against a surface [1], whereas erosive wear is caused by particle-laden fluid impacting a surface [2]. Wear-resistant materials refer to a class of fundamental materials that can resist surface material loss, scratching, spalling, and failure under such wear conditions. Their wear resistance significantly affects the load-bearing capacity, service life, and operational stability of equipment such as aircraft engines, high-speed precision cutting tools, and armored protection [3]. For a long time, the wear resistance of materials has been considered primarily controlled by hardness, and this understanding has guided scholars to focus on finding harder materials as wear-resistant materials [4]. Transition metal borides [5], carbides [6], nitrides [7], and other structural ceramics have rapidly become mainstream protective materials under extremely severe wear conditions due to their ultra-high hardness, ultra-high melting points, and excellent high-temperature structural stability. Among them, transition metal monoborides, with their unique ‘zigzag’ bonding structure composed of coexisting boron-boron covalent bonds and ionic bonds [8], not only possess excellent intrinsic hardness but also offer advantages in controllable preparation processes. Compared to superhard materials with purely covalent bonds such as diamond, they are easier to produce on a large scale, making them a highly promising new type of superhard wear-resistant ceramic system.

However, with the rapid development of high-end industrial equipment technology, the comprehensive performance requirements for wear-resistant ceramics in extreme service scenarios continue to rise. Single high hardness is no longer sufficient to meet the synergistic demands for low material wear rates and long-term stable service. At the same time, traditional single-component boride (TiB, VB, CrB, WB, etc.) pure-phase ceramics have inherent critical defects such as high brittleness and low fracture toughness. Under cyclic alternating loads and friction-wear conditions, serious problems such as grain pullout, debris spalling, and three-body friction often occur, leading to premature material failure [9]. It is evident that improving the toughness of high-hardness materials is key to the wear-resistant application of boride ceramic materials. Research has shown that an effective way to address the insufficient toughness of single-phase ceramics is to introduce a heterogeneous second-phase composite modification strategy [10], such as whiskers or metallic bonding phases, to improve ceramic toughness and wear resistance through structural optimization and performance matching among multiple phases. However, this modification method has significant drawbacks: it not only significantly reduces material hardness, but the heterogeneous second phase and the boride matrix differ fundamentally in crystal structure, bonding methods, and thermal expansion coefficients, which can cause weak phase interface bonding and residual stress accumulation. Cracks can initiate under static loads, and under dynamic conditions such as reciprocating friction or abrasive particle impact, interface cracks can rapidly propagate and expand, causing interfacial debonding, micropore formation, and overall spalling failure, making it difficult to achieve improved wear stability [11]. This severely limits the engineering application of boride ceramics in the wear-resistant field.

In this context, high-entropy ceramics [12] have been introduced into the design system of wear-resistant materials. Relying on the four core effects of high configurational entropy, severe lattice distortion, atomic hysteretic diffusion, and cocktail effects, it breaks through the short board of traditional single-component materials with single performance and limited modification methods, and can realize the multi-dimensional accurate control of material microstructure and mechanical properties, bringing a new technical path for the modification and upgrading of high-performance ceramics [13]. Since the first report of high-entropy oxide ceramics, various high-entropy ceramic systems (borides, carbides, oxides, nitrides, etc.) have been synthesized successively. It is confirmed that high entropy can significantly improve the hardness, structural stability, and oxidation resistance of ceramic materials [14]. On this basis, high-entropy monoboride ceramics (HEMBs) were reported for the first time in 2020. The HEMBs (Mo_0.2_W_0.2_Ta_0.2_Cr_0.2_Ni_0.2_)B with Vickers hardness of 48.51 ± 4.07 GPa were prepared, which successfully pushed the boride ceramics into the ranks of superhard materials, and for the first time it was clear that lattice distortion and dislocation pinning were the core mechanisms of their superhigh hardness, opening the research window of HEMBs and quickly gaining the attention of researchers [15]. Subsequently, the researchers conducted a large number of studies on the controllable preparation of single-phase powders, the optimization of sintering processes, and the optimization of single-phase hardness. For instance, the successful synthesis of HEMBs powders such as (V_0.2_Cr_0.2_Ni_0.2_Mo_0.2_W_0.2_)B [16], (Mo_0.25_W_0.25_Cr_0.25_Ta_0.25_)B [17], with a hardness of 12.3 ± 0.5 GPa for (Cr_0.2_Mn_0.2_Fe_0.2_Co_0.2_Mo_0.2_)B [18], 35.7 GPa for (V_0.19_Cr_0.21_Nb_0.21_Mo_0.21_Ta_0.18_)B, 35.2 GPa for (V_0.19_Cr_0.20_Nb_0.20_Mo_0.22_W_0.19_)B, and 40.2 GPa for (V_0.19_Cr_0.19_Nb_0.22_Ta_0.21_W_0.19_)B [19]. 44.51 ± 1.23 GPa for (VNbMoWNi)B, 42.85 ± 3.74 GPa for (VCrMoWNi)B, 41.13 ± 1.83 GPa for (VTaMoWNi)B, etc. [20]. As the research progressed, the wear resistance of HEMBs was also attempted to be explored. Guo et al. [21] prepared (V_0.2_Cr_0.2_Ta_0.2_Mo_0.2_W_0.2_)B ceramic coatings with different chemical compositions and microstructures by adjusting the process, and reported a volume wear rate of 1.29 × 10^−4^ mm^3^/(N·m) at room temperature. This study demonstrated that HEMBs have excellent wear-resistance potential. More importantly, it proved that HEMBs can achieve a reduction in wear rate through their unique microstructure design. However, similar to most wear-resistant ceramics such as WC [22], this research is highly dependent on the design of a single-phase structure and has the drawback of “placing emphasis on hardness while neglecting toughness”, ignoring the decisive role of fracture toughness and crack propagation resistance in the material’s ability to resist spalling and wear. As a result, the single-phase HEMBs have excellent hardness but suffer from severe brittle spalling under heavy-load friction, and there is a significant bottleneck in the improvement of their wear resistance. However, the (VCrMoWNi)B-Ni_2_B composite ceramic coating prepared by the atmospheric plasma spraying method has a hardness that is lower than that of the single-phase HEMBs due to the presence of the soft phase Ni_2_B. Its hardness is only 33.68 GPa, but its coefficient of friction (COF) is as low as 0.13. The study on the wear mechanism reveals that this bimodal structure is bonded by a semi-coherent interface. Through mechanisms such as interface stress transfer and deformation energy absorption of the soft phase, it effectively improves the brittleness of the material. It significantly enhances the fracture toughness at the expense of a small reduction in hardness, proving the positive impact of material toughness on the wear performance of HEMBs. This also provides a solution for the problems of heterogeneous interface crack propagation and friction failure, and offers a way to achieve a significant improvement in wear performance [23]. However, this dual-phase design essentially still employs the strategy of modifying with heterogeneous second phases, which cannot completely avoid interface mismatch and performance degradation under working conditions, and thus is difficult to achieve long-term stable wear resistance. These studies indicate that achieving the coordinated improvement of comprehensive mechanical properties such as hardness and toughness in high-entropy ceramics is still a challenge in achieving high wear resistance and the long-term service of the material. Recently, Du et al. [24] proposed that constructing homologous bimodal structures is an effective strategy for achieving performance synergy optimization. They used the decomposition of Al(OH)_3_ to prepare spinel-type homologous multiphase high-entropy oxide (Mg_1/6_Ni_1/6_Co_1/6_Cu_1/6_Zn_1/6_Al_1/6_)_3_O_4_. Compared with the traditional method of enhancing wear resistance through heterogeneous second phases, both the COF and the volume wear rate have been optimized. The lowest values are 0.44 and 3.838 × 10^−4^ mm^3^/(N·m), respectively. The minor drawback is that this high-entropy oxide has both spinel and fluorite structures coexisting, and the improvement in toughness and wear resistance brought about by the difference in crystal structure is limited. Nevertheless, this result still demonstrates that the technology of adjusting the composition and controlling the lattice arrangement of materials brought about by high entropy not only offers significant advantages in terms of material hardness and structural stability, but also indicates that high entropy can be further applied to the modification design of intrinsic homologous structures. It is worth noting that the limitations associated with single-phase materials, heterogeneous second-phase addition, and homologous heterostructured bimodal strategies indicate that isostructural compositional modulation within one crystallographic prototype is required. Considering that TiB typically exists stably in the 62nd space group, VB and CrB often crystallize in the orthorhombic structure of the 63rd space group. MoB and WB usually exist stably at room temperature in the tetragonal α-MoB structure (space group I4_1_/amd). The differences in crystal structures between different elements and the variations in their affinities are effective methods for inducing element segregation [20]. Based on the solid solution adaptability of the Ti-V-Cr-Mo-W five-element refractory components, this study proposes a strategy to fabricate a novel homologous isostructural non-stoichiometric HEMB ceramic with compositionally enriched isostructural microregions (CEIMs) by precisely regulating the chemical proportions of each component. Within the identical crystal framework, locally element-enriched solid-solution microregions are formed in situ, instead of introducing heterogeneous second phases. This design provides an alternative to the conventional single-phase and heterogeneous-toughening design frameworks of conventional single-phase preparation and heterogeneous toughening, and is designed to concurrently improve toughness, hardness, and wear resistance without extrinsic second phases.

Based on the above research approach, this work used elemental metals and amorphous boron powder as raw materials and employed spark plasma sintering (SPS) to prepare homologous HEMB ceramics (TiVCrMoW)B (HEMBs-T) containing CEIMs. The microstructure, mechanical properties, and wear resistance of HEMBs-T were systematically characterized. The relationship between the homologous isostructural microregions and the strength, toughness, and wear resistance of HEMBs-T is clarified. This research effectively overcame the technical bottlenecks of single-phase HEMBs, which have high brittleness and poor wear resistance stability, as well as the mismatch of traditional heterogeneous multiphase interfaces and the subsequent performance degradation. It provides a useful theoretical basis and technical support for the structural design and engineering application of superhard, high-toughness, long-life wear-resistant high-entropy ceramics.

## 2. Materials and Methods

### 2.1. Synthesis of (TiVCrMoW)B

Ceramic powders can be synthesized by various routes, among which mechanical mixing is one of the most commonly used methods [25]. The raw materials used for this study were Mo (99.0%, 45 μm); Cr (99.9%, 75 μm); W, V (99.95%, 45 μm); Ti (99.8%, 45 μm); and amorphous B (99%, 1–5 μm) powders acquired from ZhongNuo Advanced Material Technology Co., Ltd., Beijing, China. A mixture of these powders with a molar ratio of V:Cr:Mo:W:Ti:B = 1:1:1:1:1:5 was placed in a Teflon jar for ball milling using ethanol and zirconia balls as the milling and grinding media, respectively. An additional 5% B was added to compensate for the loss of the B element. The mass ratio of balls to powder is 2:1. Wet ball milling was performed in a planetary ball mill at 300 r/min for 12 h to achieve macroscopic uniform mixing of multi-component powders. After ball milling, the mixed slurry was dried using a rotary evaporator at 40 °C and −0.1 kPa. The dried powder was sieved with a 200-mesh standard sieve to break up agglomerated particles and obtain the powder for sintering. The mixed powder was prepressed into a circular pellet with a diameter of 40 mm. Then, the green pellet was placed in a high-purity graphite mold lined with ceramic for in-situ SPS sintering. BN was coated on the inner wall to prevent the sample from directly contacting the graphite mold and to facilitate demolding. The sintering schedule was set with a heating rate of 100 °C/min up to a maximum temperature of 1900 °C, under a constant uniaxial axial pressure of 30 MPa. An isothermal dwell of 10 min was maintained at the peak temperature, after which the specimen was allowed to cool naturally to room temperature inside the furnace. After sintering, the surface of the sample was thoroughly ground to remove the surface layer that might be contaminated by the mold and the BN coating.

### 2.2. Structural Characterization and Performance Testing

The sintered samples were polished using a diamond grinding paper, followed by phase analysis and performance testing. An X-ray diffractometer (XRD, D8 Advance, Bruker, Billerica, MA, USA) equipped with a Cu target radiation (*λ* = 0.15406 nm) was used for phase analysis. The obtained XRD patterns were refined and fitted using the Rietveld method to analyze the crystal structure, space group, lattice parameters, etc. The volume density of the sintered samples was measured by the Archimedes’ method. The relative density was obtained by comparing the volume density with the theoretical density, where the theoretical densities were calculated from the ideal stoichiometry and the lattice parameters measured by XRD. The microstructure morphology and elemental distribution of the polished surface and the surface of the worn track were characterized by using a field emission scanning electron microscope (SEM, Zeiss Sigma 300, Carl Zeiss AG, Oberkochen, Germany) combined with an energy-dispersive spectrometer (EDS). The microstructure and elemental distribution of the samples were analyzed using the transmission electron microscope (TEM, Tecnai G2 F20 FEI Company, Hillsboro, OR, USA). Subsequently, the as-sintered specimens were cut and polished. X-ray photoelectron spectroscopy (XPS, Nexsa, Thermo Fisher, Waltham, MA, USA) was used to analyze the carbon elemental composition of the surface of the sample. The Vickers hardness test was conducted using the HXD-1000TMC/LCD type microhardness tester (Shanghai Taming Optical Instrument Co., Ltd., Shanghai, China), with a uniform holding time of 15 s. The indentations were randomly distributed. Indentations located on visible pores, cracks, inclusions, abnormally large defects, or overlapping indents were excluded. CEIMs were not deliberately excluded. For each load, at least ten valid indentations were randomly distributed on the polished surface. The bending strength was measured via a three-point bending test on 2 mm × 3 mm × 16 mm bars (support span: 10 mm, cross-head speed: 0.5 mm/min). Fracture toughness was measured using the single-edge notched beam (SENB) method with 2 mm × 4 mm × 22 mm specimens (span: 16 mm, cross-head speed: 0.05 mm/min). A unilateral open-blade approach with a 0.2 mm diameter diamond wire was employed to fabricate a 2 mm deep notch at the midpoint of the 16 mm span edge. The nominal notch-tip radius is approximately 0.1 mm. Because the notch is not a sharp pre-crack, the measured value is treated as an apparent SENB fracture toughness, and no notch-radius correction was applied. Seven specimens were tested for each measurement. The detailed calculation formula is given in Section S1 of the Supplementary Information. Under room temperature and atmospheric conditions, reciprocating sliding wear tests were performed using the reciprocating friction and wear testing machine (UM T-3, Bruker, Billerica, MA, USA), and the sample size was Φ20 × 4 mm. A 6 mm diameter highly polished Si_3_N_4_ ceramic ball (surface roughness values: 0.014 μm, Hv_1_ = 1400–1700) was selected as the counterpart. The surface roughness of the polished sample was characterized by a surface roughness tester (TR100 Beijing JiTai Tech Detection Device Co., Ltd., Beijing, China). Under atmospheric conditions (34 °C and 50% relative humidity), the wear tests were performed under a normal load of 10 N with a reciprocating stroke of 5 mm. The actual physical wear-track length (*l*_track_) was 5 mm and was used for volume integration; one complete cycle corresponds to a sliding distance of 0.01 m (2 × 5 mm). The sliding frequency was set to 4 Hz, and the test lasted for 30 min, corresponding to a total sliding distance (L) of 4 Hz × 60 s × 30 min × 0.01 m = 72 m. The testing machine continuously and in real time collected the evolution curve of the COF over time. The 3D morphology of the wear tracks and the two-dimensional curves of the cross-sections of the wear track were observed using a white light interferometer microscope (RTEC lambda, CA, USA). For the representative wear test (Exp. 1), two-dimensional cross-sectional profiles were collected at four equally spaced locations along the wear track. To evaluate repeatability, two additional independent wear tests (Exp. 2 and Exp. 3) were performed under identical conditions. For each of these two repeat tests, six equally spaced cross-sectional profiles were collected (six positions per repeat test). For each experiment, the wear volume was obtained by integrating the cross-sectional area below the original reference plane along the 5 mm physical wear-track length. The volume wear rate *W* was calculated using Formula (1) [26]

*W* = *V*/(*F* × *L*) (1)

where *V* is the volume loss, *L* is the sliding distance, and *F* is the normal load.

## 3. Results

### 3.1. Phase and Structure Analysis

**Figure 1:**
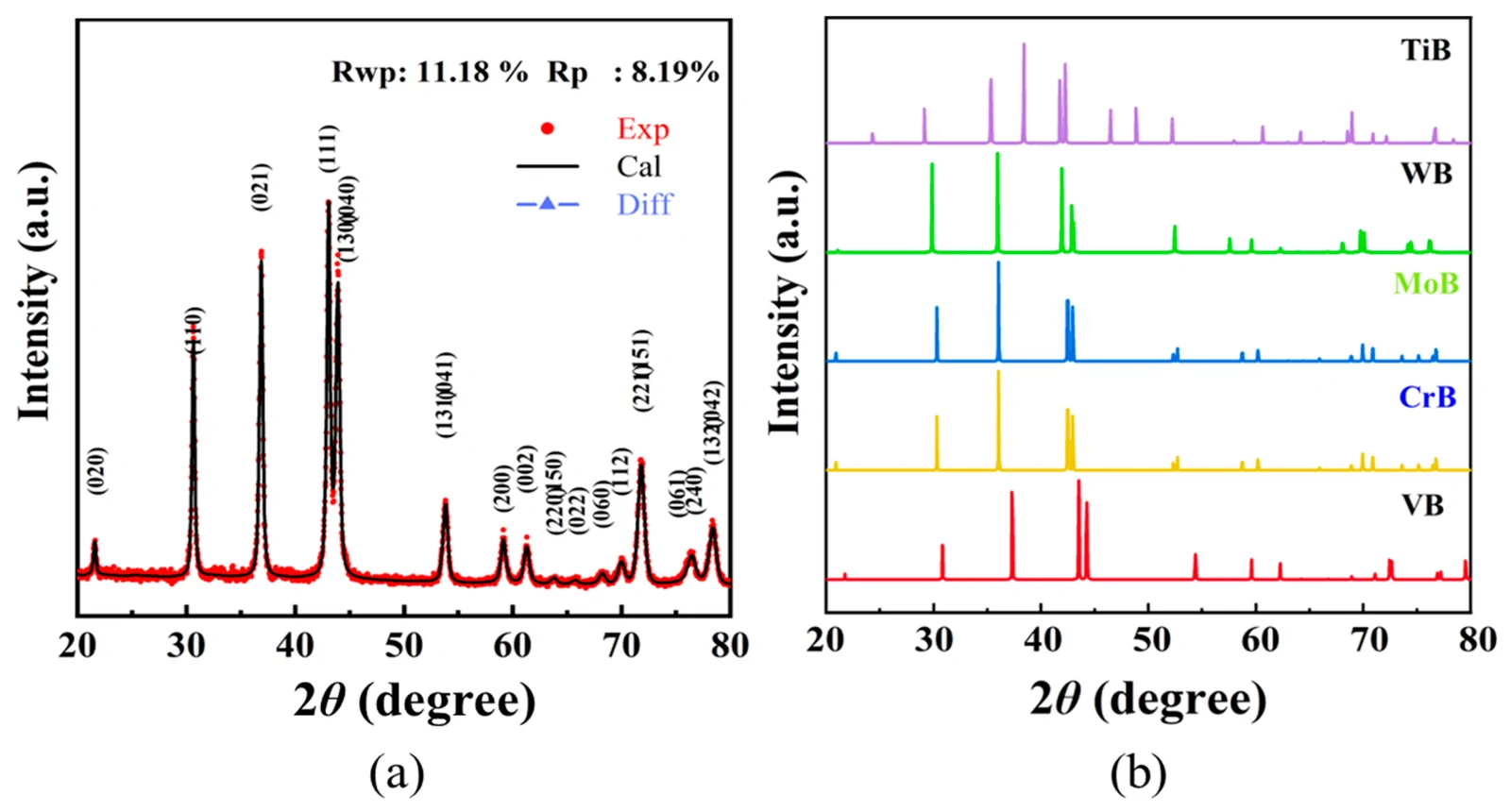
(**a**) XRD pattern of the prepared HEMBs-T and (**b**) the XRD patterns of the theoretical calculation of a single-component monoboride.

XRD characterization was first conducted to analyze the phase composition of the as-prepared sample synthesized at 1900 °C for 10 min, and the results are shown in Figure 1. The XRD patterns show that all the diffraction characteristic peaks of the prepared samples are consistent with those of the orthorhombic Cmcm-type CrB. No obvious characteristic diffraction peaks of a second phase or diffraction peak splitting were found, presenting a typical single-phase diffraction pattern. This indicates that the five metal atoms were solid-dissolved with each other after SPS sintering, forming a multi-component solid solution that inherited the orthorhombic crystal structure of CrB. To further verify the formation of the solid solution and obtain precise crystallographic information, the XRD patterns were refined using the Rietveld method. The reliability factors R_wp_ and R_p_ of the refinement were 11.18% and 8.19%, respectively, both of which were lower than the 15% threshold for credibility, indicating that the refinement results were reliable [27]. As listed in Table 1, Rietveld refinement gives lattice parameters of a = 3.12 Å, b = 8.23 Å, and c = 3.00 Å. The lattice constants differ from those of individual orthorhombic single TiB, WB, MoB, CrB, or VB, is close to the average lattice parameters of the five monoborides, which is conforming to the typical characteristics of random solid solution of atoms with different radii. Based on this result, the relative density of the HEMBs-T is 94.13%. Figure 1b further compares the experimental diffraction profile with the standard diffraction peaks of orthogonal TiB, WB, MoB, CrB, and VB. It can be observed that the diffraction peaks of HEMBs-T are similar to those of five single-component monoborides, without the peak position superposition or splitting phenomenon caused by multiphase mechanical mixing, confirming the formation of multi-component solid solutions. Furthermore, the strongest diffraction peak corresponds to the crystal plane (111), and there is a significant broadening of the peak. This is due to the significant lattice distortion caused by the significant difference in atomic radii among the five metal atoms of V, Cr, Ti, Mo, and W, which not only causes the lattice constant to deviate from that of a single compound, but also leads to a noticeable broadening of the diffraction peak.

**Figure 2:**
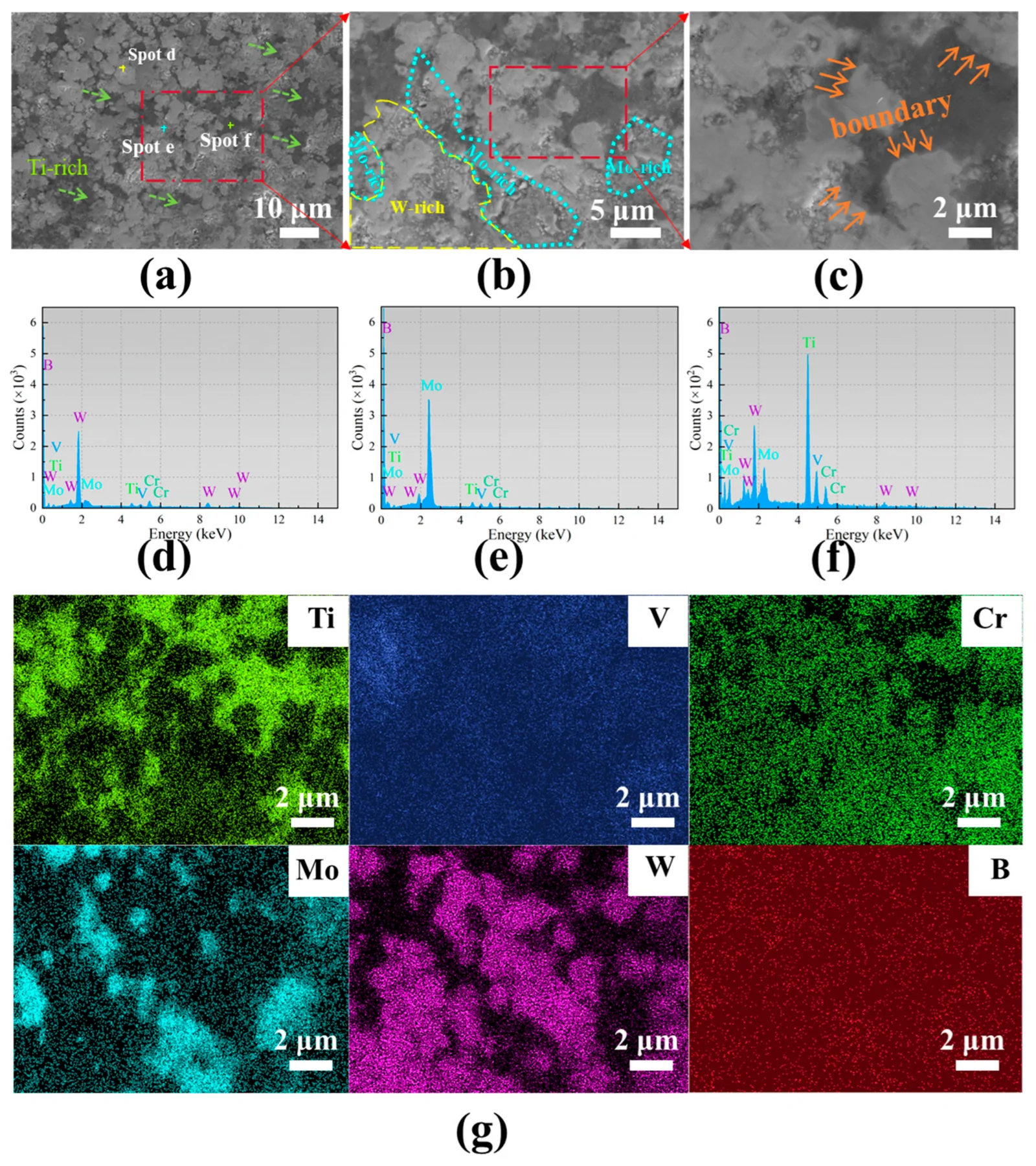
(**a**–**c**) SEM images of the polished surface of HEMBs-T by SPS sintering at 1900 °C for 10 min, and (**d**–**f**) EDS spectra from point scans, along with (**g**) the corresponding EDS mapping of Ti, V, Cr, Mo, W, and B elements. (The green arrow points to the Ti-enriched area, the orange arrow indicates the interface between different microregions; the area circled by the yellow curve is the W-enriched area, and the area circled by the cyan curve is the Mo-enriched area).

The SEM images of the polished surface of the HEMBs-T and the corresponding EDS mapping of Ti, V, Cr, Mo, W, and B elements are shown in Figure 2. From the morphology image of the polished surface shown in Figure 2a, it can be clearly seen that the prepared HEMBs-T has a compositional contrast (dark gray, light gray, and bright silver microregions), and these microregions are uniformly distributed. No abnormally coarse grains demonstrate good microstructure uniformity. The grain size is concentrated in the range of 2–5 μm, which is significantly smaller than 8.03 ± 2.77 μm of (V_0.2_Cr_0.2_Mo_0.2_W_0.2_Ni_0.2_)B prepared by SPS sintering at 1900 °C and 30 MPa for 10 min [16], 14.7 ± 9.0 μm of (V_0.2_Cr_0.2_Nb_0.2_Mo_0.2_Ta_0.2_)B, 18.9 ± 14.6 μm of (V_0.2_Cr_0.2_Nb_0.2_Mo_0.2_W_0.2_)B and 13.4 ± 7.9 μm of (V_0.2_Cr_0.2_Nb_0.2_Ta_0.2_W_0.2_)B prepared by SPS sintering at 50 MPa for 10 min [19]. Furthermore, the number of pores in the sample is extremely low; only a small number of isolated closed pores exist at the grain boundaries, which is in good agreement with the relative density of 94.13% measured by the Archimedes method. The high relative density can weaken the stress concentration caused by pores and the initiation of microcracks under external forces. In order to clarify the composition changes between different phases, EDS analysis was conducted at spots d, e, and f on the three phases, respectively. The corresponding results are shown in Figure 2d–f. It can be seen that spot d is dominated by characteristic peaks of W, while spot e displays major Mo signals, and spot f exhibits prominent Ti characteristic peaks. Furthermore, in the three-spot EDS analysis, peaks of five metals were all detected, indicating that the three representative microregions belong to the W-rich, Mo-rich, and Ti-rich solid-solution microregions, respectively. The quantitative detection results of elements at the three points are shown in Table S1. It can be seen that the elemental ratios at different locations are different, showing phenomena rich in Ti, Mo, and W, respectively. Moreover, the composition of the metal sublattice in these three regions obviously deviates from the constant molar ratio, and the significant difference in element content indicates that they are microregions of isostructural solid solutions with different compositions, rather than small fluctuations in a single solid solution. To more clearly distinguish the distribution of the CEIMs and the distribution of each element, the element distribution on a selected local region shown in Figure 2b was characterized. The result reveals a clear difference in elemental distribution (Figure 2g). V and B are homogeneously distributed throughout the microstructure, while Cr-Ti and Mo-W pairs exhibit poor coexistence, resulting in the formation of Ti-rich, Mo-rich, and W-rich regions. Despite local composition fluctuations, these microregions are well combined, as shown in Figure 2c. The absence of obvious interface cracks and debonding indicates that the different microregions are not simply physically stacked. Combined with the results of the single-phase diffraction peaks presented by XRD, it can be indicated that the prepared HEMBs-T contains three representative types of CEIMs, all sharing the same orthorhombic monoboride framework. It should be pointed out that binary single borides differ greatly in Vickers hardness and elastic modulus, so the Ti-rich, Mo-rich, and W-rich microregions may exhibit different local mechanical responses because of their different compositions. These microregions are still five-component solid solutions, and their local properties are not equivalent to those of the corresponding binary borides. This has been supported by the work of Wang et al. [33], which showed that composition fluctuations formed by intrinsic elemental segregation can alter the local hardness and softness without adding a second phase. Such composition fluctuations may generate an in-situ interleaved microstructure of soft and hard microregions, providing a possible microstructural basis for the mechanical and tribological performance. However, limited by the inability to directly collect the local hardness of element-segregation microregions, the local hardness differences among these microregions remain hypothetical. The reported Vickers hardness represents the macroscopic composite response of the heterogeneous microstructure. Similar composition-fluctuation effects have been reported in the regulation of mechanical properties of high-entropy materials [34].

**Figure 3:**
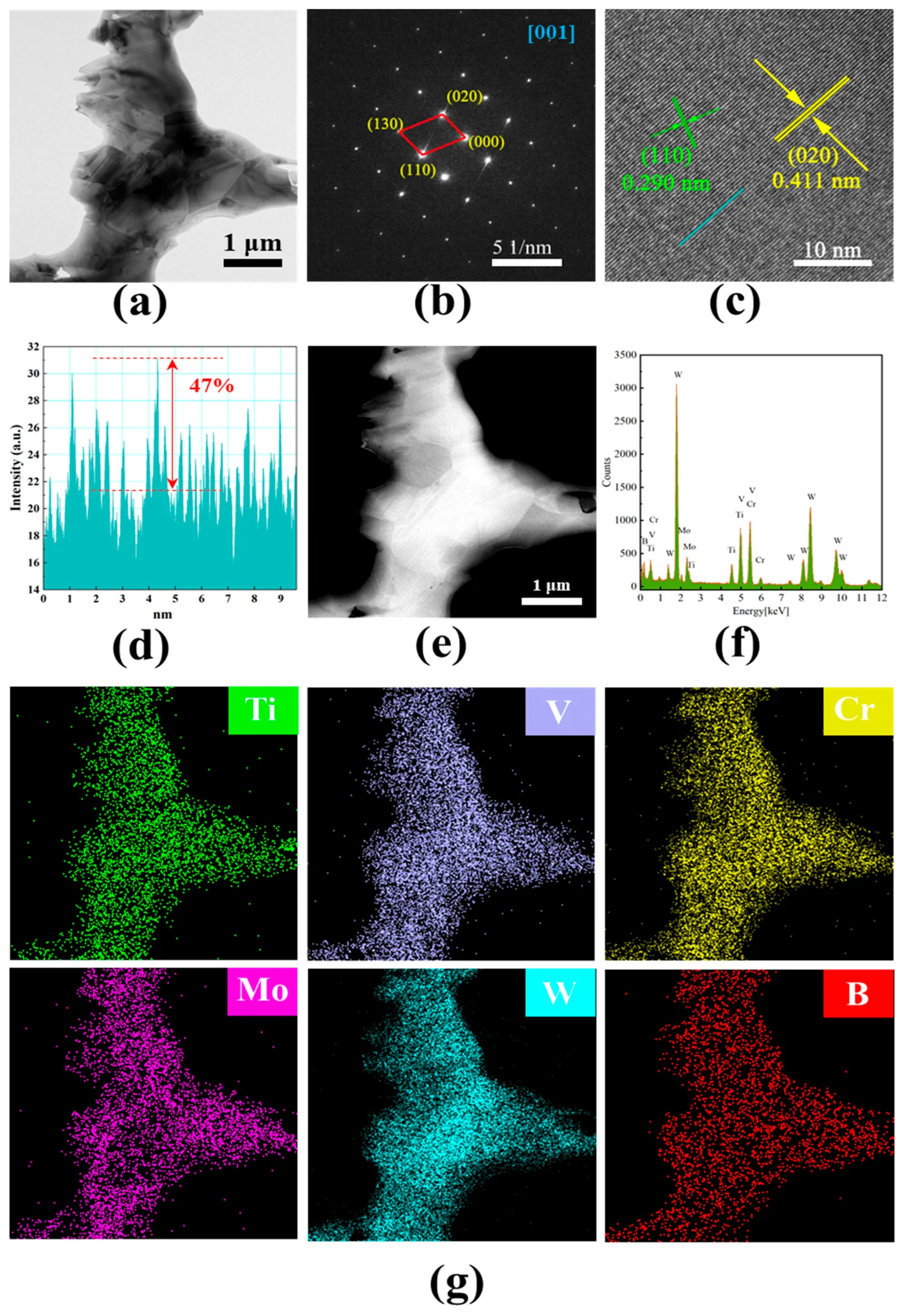
TEM analysis of the HEMBs-T: (**a**) TEM image, (**b**) SAED pattern, (**c**) HRTEM image, (**d**) atomic column brightness difference, (**e**) HAADF, (**f**) EDS energy spectrum, (**g**) EDS compositional maps.

In order to further analyze the phase composition and microstructure of the prepared HEMBs-T, TEM analysis and corresponding EDS analysis were employed to investigate the crystal structure and chemical uniformity of the HEMBs-T. The results are presented in Figure 3. The typical grain morphology of the HEMBs-T can be clearly observed by TEM, where the grains are closely bonded together (Figure 3a). In addition, the selected area electron diffraction (SAED) of the synthesized HEMBs-T exhibits clear and regular diffraction spots, corresponding to the [001] zone axis. These indexed diffraction spots indicate that the HEMBs-T belong to a well-defined single-phase structure, as evidenced in Figure 3b. The results are highly consistent with the orthogonal structure shown by XRD. The high-resolution transmission electron microscopy (HRTEM) image is shown in Figure 3c, from which we could intuitively observe the phenomenon that periodic crystal structures were observed, with interplanar spacing of 0.290 nm and 0.411 nm, respectively, which were consistent with (110) and (020), respectively. The background intensity analysis of a randomly selected atomic column shows that the difference between the maximum and minimum intensities can be up to 47%, revealing the random distribution of different metal atoms [35], as shown in Figure 3d. Figure 3e shows the high-angle annular dark field (HAADF) images, and the EDS spectrum is shown in Figure 3f. It is evident that all elements have been detected. Additionally, as shown in the elemental maps in Figure 3g, metal W exhibits a slight segregation, while the other elements are uniformly distributed. These findings are highly consistent with the previous SEM results, demonstrating the occurrence of selective elemental segregation in this material. This local compositional segregation is not caused by a structural phase transformation, but is instead the result of the combined influence of atomic diffusion rate differences and chemical affinity, leading to microregions’ component fluctuations [36]. In addition, the surface carbides and core-level binding energies of the samples are examined using XPS; the detailed results have been presented in Figure S1. XPS analysis showed that the C 1s signal was dominated by amorphous carbon at 284.8 eV. There is no obvious metal-carbon characteristic peak on the polished surface.

It should be noted that due to the low X-ray production yield and limited surface sensitivity of B and C and other light elements, EDS and XPS have semi-quantitative characteristics for the quantitative analysis of these elements. Therefore, the B content is reported as the nominal value. Secondly, the Rietveld refinement method is more sensitive to the average values of crystal structure and lattice parameters, but not to the occupation situation and vacancies of light elements. Therefore, the chemical stoichiometry of B cannot be precisely determined. Thirdly, the detection limit of XRD for secondary phases is usually about 1–5 wt%; SAED samples can only detect local nanoscale regions, while the XPS probe can only detect a region of approximately 10 nm near the surface, and cannot rule out the possibility of the presence of a small number of carbides in the sample block. Therefore, substances below these detection limits cannot be excluded. Therefore, affected by the detection limits, the term “single phase” in this work refers to the single phase within the detection limits of XRD and SAED, and trace second phases below these limits cannot be completely excluded.

### 3.2. Mechanical Properties Analysis

**Figure 4:**
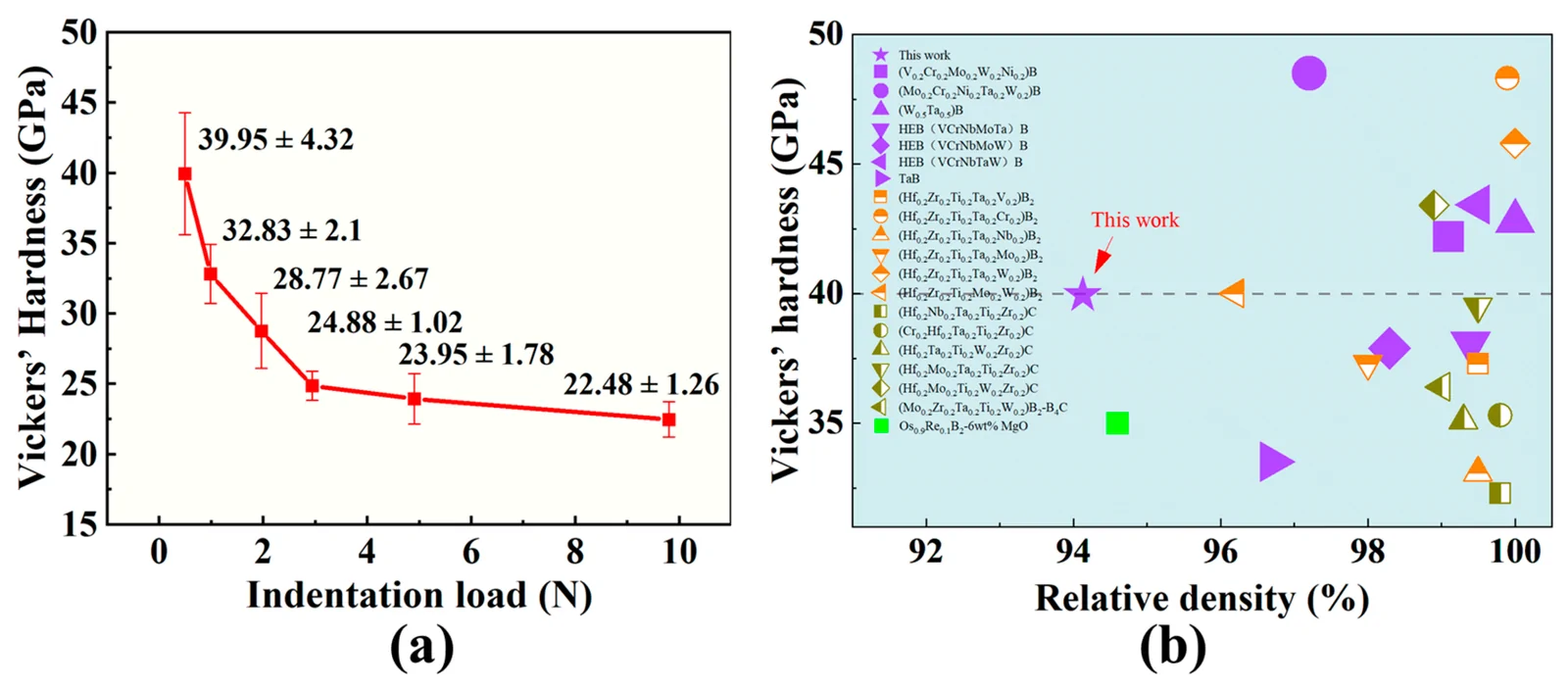
(**a**) Measured Vickers hardness of HEMBs-T. (**b**) Scatter plot of Vickers hardness of different specimens under 0.49 N load from the literature [15,19,38,42,43,44] and this work at different relative densities.

The hardness of HEMBs-T prepared by sintering was measured using a Vickers hardness tester and is presented in Figure 4a. Figure 4a shows the Vickers hardness of HEMBs-T measured under different indentation loads (0.49 N to 9.8 N). It is obvious that as the load increases, the Vickers hardness decreases. At a load of 9.8 N, the Vickers hardness was 22.48 ± 1.26 GPa, while at a load of 0.49 N, the hardness reached 39.95 ± 4.32 GPa. This load-hardness relationship (i.e., indentation size effect) has already been demonstrated in previously reported high-entropy ceramics [18]. It is commonly observed in materials with high hardness, brittleness, and poor compressibility. This phenomenon is mainly attributed to the elastoplastic coupling behavior during plastic deformation and the friction effect during the indentation process [19]. Under low load conditions, the indentation and plastic regions are smaller, and the plastic deformation is easier to be confined to a single grain or a single component-enriched microregion; The grain boundary slip and microcrack propagation are not activated, so the measured hardness is high. However, the measured values may also reflect the local response of individual microregions and may be sensitive to local Ti/Mo/W compositional enrichment. However, this local effect is inferred rather than directly measured (see Section 3.1), and it may contribute to the relatively large standard deviation. With the increase in load, the indentation size increases; the indentation covers multiple micro regions and grain boundaries, and microcracks and intergranular fractures may occur. The measured values tend to be close to the average response of multiple micro regions, and the standard deviation decreases accordingly. The measured results are closer to the comprehensive hardness performance of the material matrix [37]. Unfortunately, the measured Vickers hardness of the present material is 39.95 ± 4.32 GPa under a load of 0.49 N and doesn’t exceed the artificially defined superhard performance standard commonly used in previous studies on superhard borides, which is 40 GPa under a 0.49 N load [38]. Nevertheless, this value is significantly higher than that of most high-entropy diborides, high-entropy carbides, and conventional Ni-free monoborides (Figure 4b) and is only slightly lower than those of Ta- and Ni-containing noble-metal systems. However, under the equivalent high-load condition, HEMBs-T reaches 22.48 ± 1.26 GPa at 9.8 N, which is equivalent to the high-load hardness of equimolar HEMBs reported in the literature [19] (about 22–26 GPa at 9.8 N). It is worth noting that this inference should be examined in the context of the existing studies on HEMBs. Although the equimolar single-phase system has established a high hardness standard, the fracture toughness and wear rate have not been reported under the same tribological conditions. In addition, due to the fact that the preparation of equimolar single-phase high-entropy ceramics with the same composition will inevitably introduce different sintering heat histories, the effects of density and grain size cannot be separated independently, resulting in the inability to achieve the same conditions. The single-phase XRD patterns (Rwp = 11.18%) refined by Rietveld still show that the heterogeneity belongs to the composition level rather than the structure level, which cannot be achieved by the heterogeneous second-phase addition method.

**Figure 5:**
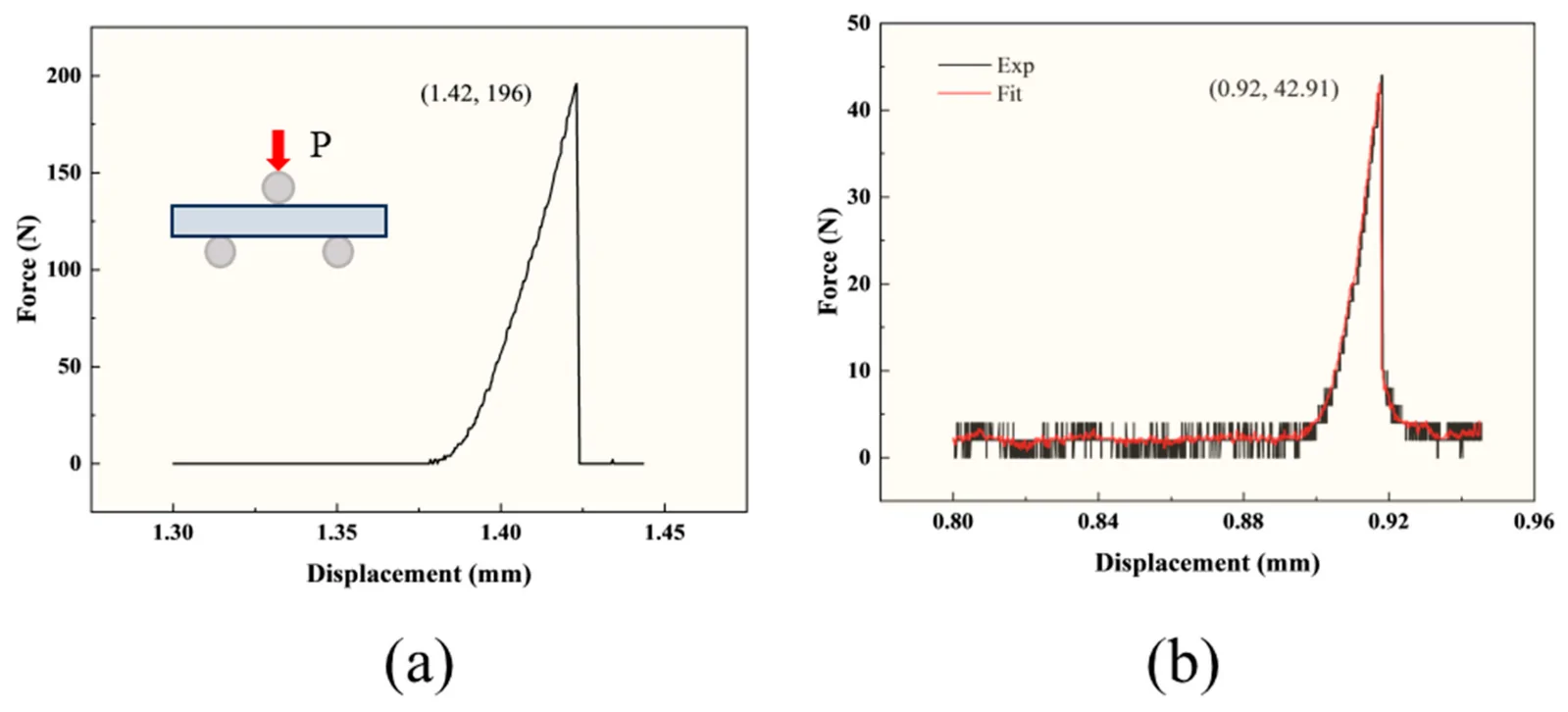
Typical load-displacement curves of HEMBs-T; (**a**) three-point bending test, and (**b**) SENB test.

In addition to hardness, the fracture strength and toughness of HEMBs-T were also tested and analyzed. The results are listed in Table 2. The flexural strength is 243.3 ± 2.4 MPa, which is comparable to those of high-hardness or even superhard high-entropy carbides (TiVNbTaMo)Cx (256 ± 32 MPa) [39] and (Zr, Ti, Nb, Ta, Hf)C_0.8_ (218 ± 33 MPa) [40]. The typical force-displacement curve of the three-point bending test was plotted in Figure 5a, which displays that the load of the material changes approximately linearly with the displacement. Upon loading to the peak load of 196 N, the load suddenly dropped vertically, and the specimen broke instantly. No obvious yield deformation, steady crack propagation, or a slow load decrease stage was observed in the curve, demonstrating typical brittle fracture characteristics. This suggests that before the external load reaches the critical bending stress, the material mainly undergoes elastic deformation, and there is no significant damage evolution or energy dissipation mechanism within the material. Figure 5b shows the load-displacement curve obtained from the fracture toughness test of the single-edge notched beam (SENB). To better analyze the mechanism behind the curve, the experimental curve is fitted. It can be observed that as the displacement increases, the stress rapidly rises to the peak load of 42.91 N, followed by a sharp drop of the load back to the baseline level. There was no obvious step-like crack propagation in the curve, indicating that the material’s ability to absorb fracture energy was still limited [41]. This confirmed that the fracture process of HEMBs-T was still dominated by brittle failure. However, the measured fracture toughness reaches 3.39 ± 0.36 MPa·m^1/2^ (Table 2). This apparent SENB toughness is comparable to or higher than the values reported for related (V_0.2_Cr_0.2_Mo_0.2_W_0.2_Ni_0.2_)B systems [16]. However, because the present value is an apparent SENB toughness without notch-radius correction, this comparison is qualitative. The result therefore suggests that the CEIMs architecture may contribute to toughness improvement. The value estimated from the representative curve in Figure 5b (~3.62 MPa·m^1/2^) corresponds to a single specimen and is presented for illustrative purposes only. The reported fracture toughness of 3.39 ± 0.36 MPa·m^1/2^ is the mean ± standard deviation of seven valid SENB specimens. Although they do not have the same chemical composition, this toughness level still shows the contribution of the unique CEIMs in this study to the improvement of fracture toughness without introducing. It should be noted that the 0.2 mm diamond-wire notch has a finite tip radius (nominal r ≈ 0.1 mm). Thus, the notch cannot be regarded as an ideal sharp crack. A blunt notch reduces the stress concentration at the notch root and generally requires a higher load for crack initiation, which tends to overestimate the apparent toughness relative to a pre-cracked specimen; machining-induced microcracks and residual stress may partly offset this effect. Since no notch-radius correction was applied, the value of 3.39 ± 0.36 MPa·m^1/2^ is reported as an apparent SENB toughness (*K_IC_*, app), and comparisons with literature toughness values are qualitative.

### 3.3. Sliding and Wear Properties

**Figure 6:**
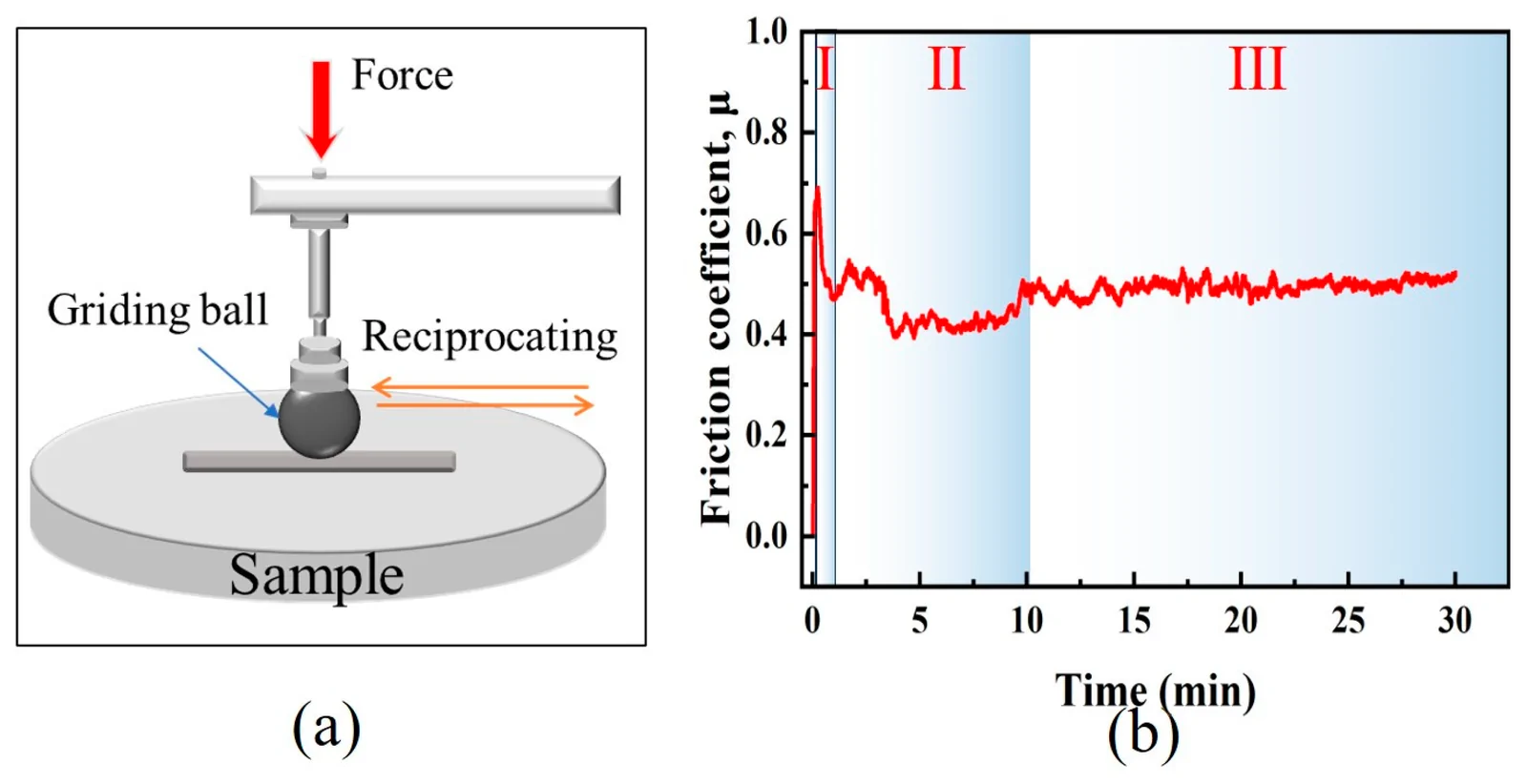
(**a**) Schematic diagram of friction and wear; (**b**) Typical curve of COF.

The friction behavior of the composite ceramics was studied in the form of reciprocating sliding friction and mainly analyzed using the COF and volume wear rate. Before the friction test, all samples were polished step by step with diamond abrasive paper. After polishing, the surface roughness of the sample is 0.58 ± 0.05 μm, and the detailed results are shown in Table S2. A schematic diagram of the reciprocating friction test is shown in Figure 6a, and the obtained friction curve is displayed in Figure 6b. The COF curve of HEMBs-T can be divided into three typical stages according to its evolution with sliding time. The first minute of the friction process is the running-in stage. During this stage, the micro-convexities on the original surface of the sample had point contact with the grinding balls, resulting in high contact stress. This high contact stress caused the micro-ridges to exhibit locking and brittle micro-cracking behaviors, leading to a rapid increase in the COF and the formation of a distinct initial peak, with the maximum COF reaching approximately 0.70. Friction enters the running-in transition stage within 1 to 10 min. During this stage, the sharp micro-convex body on the surface is crushed and stripped, and a large number of wear debris enters the friction interface. The wear mechanism gradually changes from two-body wear to three-body wear. As a result, the COF slowly decreases, and the friction interface gradually becomes smooth. The non-uniform morphology of the wear track along the sliding direction suggests non-uniform local removal among microregions with different hardness and fracture toughness. The stepwise fluctuation of the COF corresponds to the generation–compaction–expulsion process of debris at the contact interface, consistent with the formation of a friction layer. After 10 min of friction, due to the continuous removal of loose grinding debris and the decrease in the generation level of debris at the interface, the friction contacts gradually shifted from being dominated by loose abrasive particles to direct contact between the friction layer and the grinding balls, resulting in a slight increase in the COF. Finally, the generation rate of wear debris at the wear tracks and the loss rate reached a dynamic equilibrium, forming a continuous and stable interface for wear debris transfer, and the friction entered the steady-state stage. As a result, the fluctuation range of the COF was extremely small, remaining basically stable at 0.52. This indicates that the composite material can form a stable friction interface after the running-in stage and exhibits stable friction behavior within the 30 min test duration. The step-like change of the curve also confirms the inherent characteristic of materials with different mechanical properties of the composite phase components.

**Figure 7:**
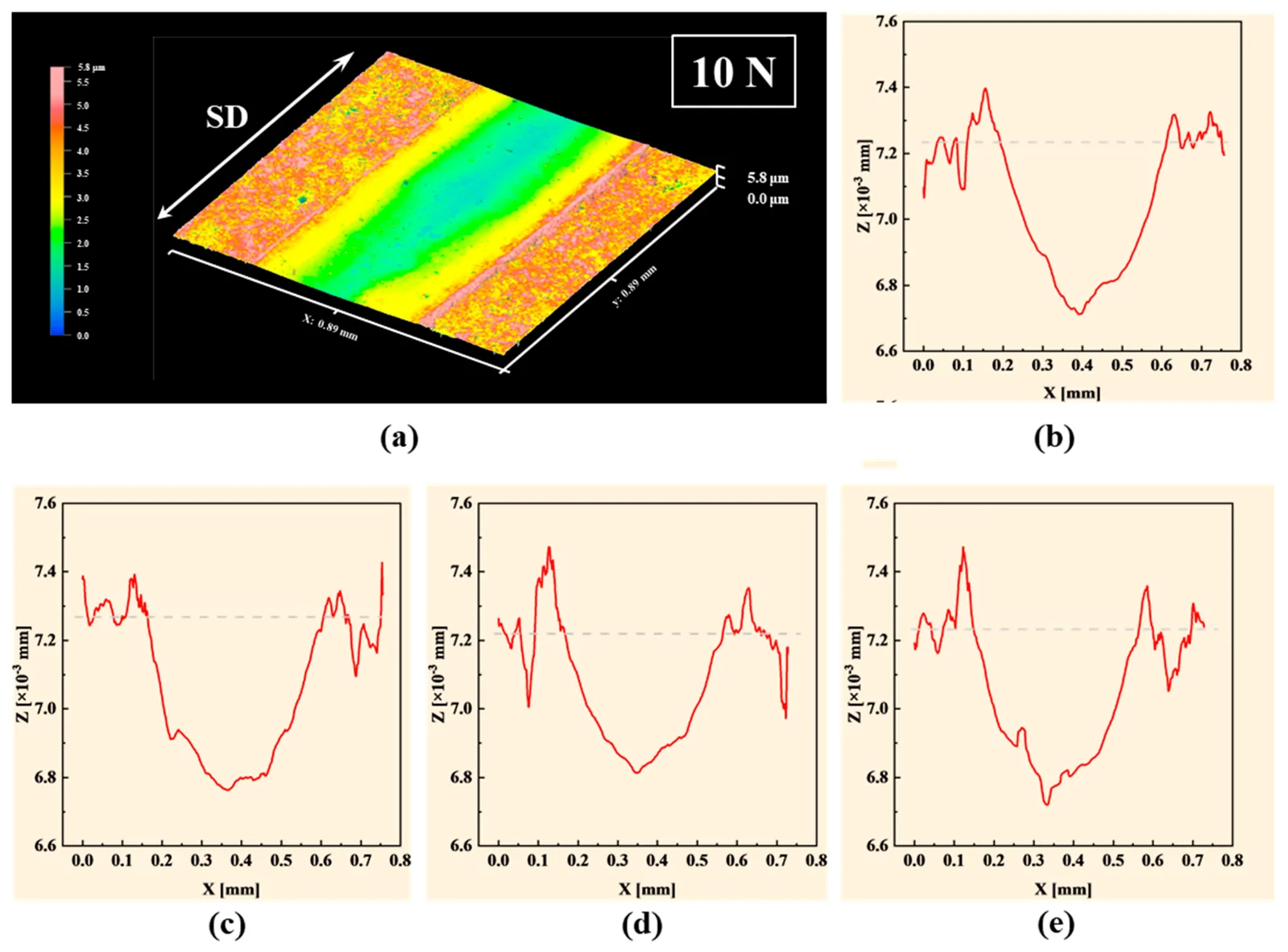
(**a**) 3D morphology of wear surface of the composite material under 10 N loads. (**b**–**e**): 2D cross-sectional profile at different positions along the x direction. (SD: sliding direction; The dashed lines in Figure 7b–e represent the original reference plane of the sample).

To observe the wear condition of the worn area, the three-dimensional surface morphology of the wear tracks on the ceramic composite material was measured and shown in Figure 7, where SD represents the reciprocating sliding direction. The long strip-shaped wear tracks formed by reciprocating friction can be clearly observed. At the same time, it can be noticed that material removal occurs at the wear tracks, thereby forming depressions. The edges on both sides of the wear tracks show obvious bulges, which is due to the accumulation of the worn debris that was compressed and migrated at the boundaries of the wear tracks during the friction process. Furthermore, the color gradient in the worn area shows that there are some surface bumps and dips inside the wear tracks, suggesting that the bottom of the wear tracks is not absolutely flat but rather has local concave-convex and micro-peeling features. Figure 7b–e show the transverse cross-sectional profile curves at different positions of the wear tracks. All cross-sectional profiles present typical U-shaped features, where the dashed line represents the reference height of the original surface. It can be observed that the removal of the material causes a significant drop in the height of the center area of the wear track. The positions on both sides of the wear track contour are higher than the original reference surface, further confirming that there is a phenomenon of abrasive debris accumulation at the edge of the wear track. This is the result of hard abrasive debris that flakes off during reciprocating friction being subjected to repeated shear compression by the grinding ball, moving to the sides of the wear track and then being re-pressed and accumulated. By comparing the cross-sectional contours of the four positions in the representative wear test (Exp. 1), it can be observed that the maximum wear depth of the wear tracks at different test positions shows a slight fluctuation, indicating that the wear damage of the composite material along the sliding direction has certain non-uniformity. This phenomenon is caused by the differences in mechanical properties among the various components of the CEIMs, as well as the uneven distribution of these components. During the friction process, local preferential peeling of microregions and random plowing action of tri-body abrasive particles occur, resulting in inconsistent removal degrees at different positions of the wear tracks. At the same time, the bottom of the cross-section shows slight wave-like bumps and dips, suggesting that there are plow grooves and local material peeling pits inside the wear tracks. This indicates that the wear process involves both abrasive wear and local brittle peeling mechanisms. The wear debris not only participates in the construction of the friction transfer layer at the friction interface, but some of it is also pushed to the edge of the wear track to accumulate. This matches the dynamic process of ‘generation-compaction-expulsion’ of wear debris reflected in the COF curve mentioned earlier. From the 2D cross-sectional profiles of the wear tracks, the wear volume was estimated for each of the four positions by measuring their cross-sectional areas (Table S3). The calculation process of the wear volume is shown in the Supplementary Information. Notably, the integration for wear volume calculation was performed only on the cross-sectional area beneath the original specimen reference plane. To evaluate the intrinsic wear resistance of the material and experimental repeatability, two additional independent repeated tests were carried out under identical experimental conditions, and the obtained wear volumes are listed in Table S4. It should be noted that the representative wear test used four cross-sectional profiles (Table S3), whereas each of the two additional repeat tests used six profiles (Exp. 2 and Exp. 3 in Table S4). Based on the three sets of volume wear rate results, the mean volume wear rate is as low as (1.11 ± 0.4) ×10^−6^ mm^3^/(N·m), with the lowest single-test value being 6.94 × 10^−7^ mm^3^/(N·m). This average value is lower than 4.11 × 10^−6^ mm^3^/(N·m) of high-entropy diboride ceramics under identical test conditions [45], and slightly higher than that of high-entropy carbides reported in the literature [46] under the same conditions. However, the comparison is not under identical conditions: Ref. [45] used a 60 min sliding test, whereas the present work used 30 min. Even with the same normal load and counterpart material, differences in test duration and total sliding distance may affect the wear mechanism and the measured wear rate.

**Figure 8:**
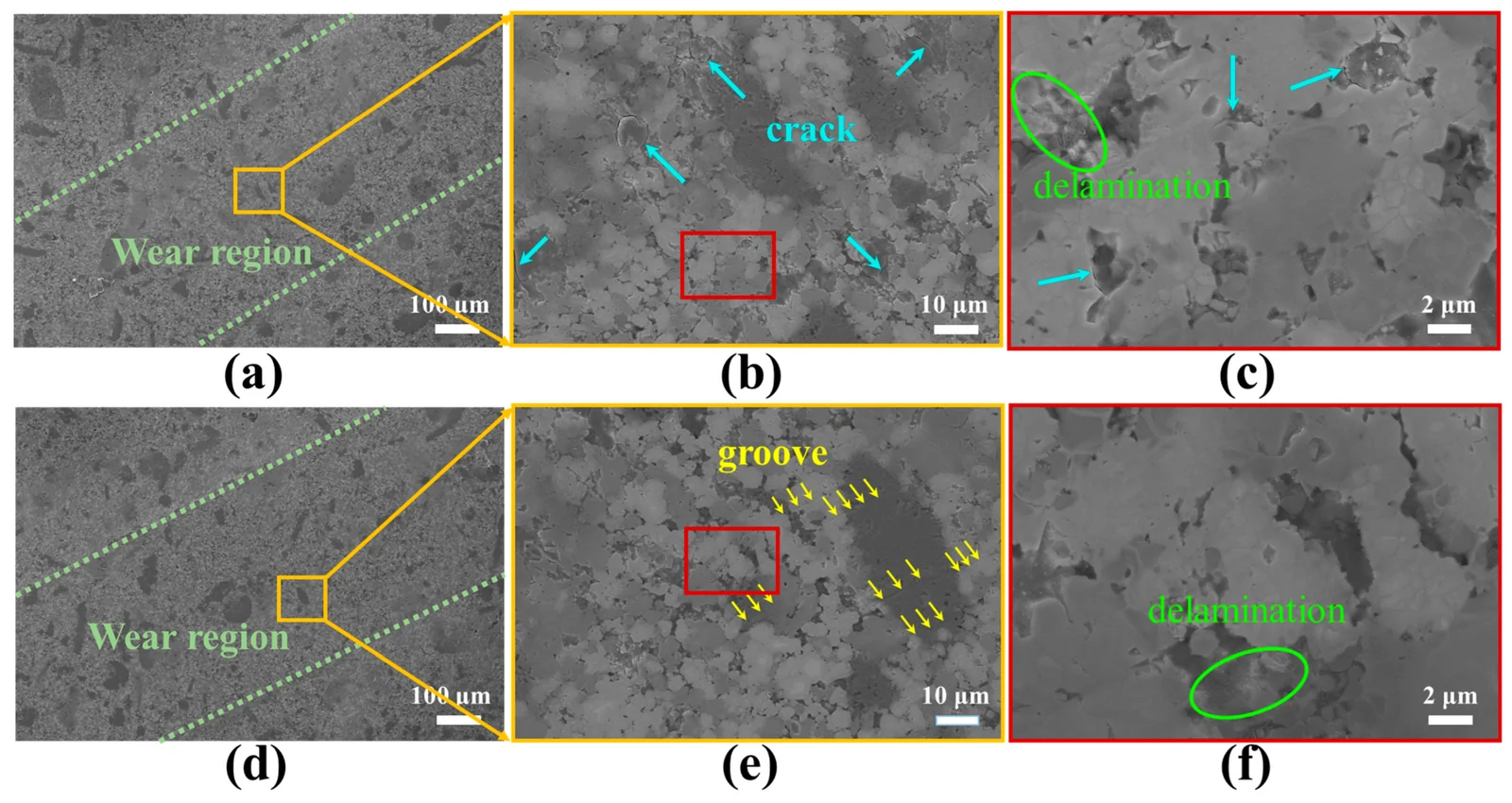
SEM characterization of wear tracks of HEMBs-T under an applied normal load of 10 N. (The large cyan arrow represents a microcrack, and the small yellow arrows indicate the grooves. The red box represents the area of the local zoomed-in view, and the corresponding enlarged images are shown in Figure 8c and Figure 8f, respectively).

By characterizing the surface morphology of different areas of the wear tracks after the reciprocating friction, the corresponding wear mechanism was systematically analyzed. Continuous long strip-shaped wear areas were formed on the surface of the sample, and there was no obvious crack initiation or propagation at the edge of the wear tracks (Figure 8a,d). However, the initiation and propagation of microcracks were observed at the boundary between microregions (Figure 8b), yet no macroscopic cracks penetrated the matrix, indicating that the initiation of microcracks at the interface is one of the main forms of friction failure of this material. By magnifying the interface area, the micro-area with layered peeling characteristics can be observed (Figure 8c), indicating that cyclic alternating frictional stress can also cause continuous fatigue damage at the interfaces between different microregions, ultimately leading to slight flaky spalling on the material’s surface layer. This improved resistance to crack propagation and fatigue damage is mainly attributed to the unique advantages of a multicomponent solid solution structure that has the same crystal type. The significant lattice distortion induced by the multi-atom substitution within the material can effectively pin down dislocations and hinder their sliding movement. Meanwhile, the slight lattice mismatch at the boundaries between different CEIMs caused by chemical composition fluctuations can blunt microcracks to some extent, may retard the rapid propagation and penetration of cracks, and improve the inherent brittleness and fatigue defects of monoborides. Further magnification of the worn area (Figure 8e,f) reveals a typical groove that has grinding marks, demonstrating that abrasive wear occurs in the early stage of the friction process and is one of the core wear mechanisms of this composite material. At the same time, it has been found that the wear-rate range of unlubricated sliding contact is about 10^−7^–10^−2^ mm^3^/(N·m) for adhesive wear and 10^−5^–10^−1^ mm^3^/(N·m) for abrasive wear [47]. The wear rate of this material is (1.11 ± 0.4) × 10^−6^ mm^3^/(N·m), which falls in the low-value region of the adhesive wear range and is below the lower bound of the typical abrasive wear regime. It implies that the wear damage of this material has not yet fully entered the adhesive wear-dominated stage. It is highly consistent with the results of this study. Overall, the prepared HEMBs-T did not exhibit severe brittle fractures during the friction process. The wear damage was mainly manifested as slight scraping wear, the initiation of interface microcracks, and local layered peeling. This mild wear behavior is highly consistent with the extremely low volume wear rate, and the steady-state COF remains stable over the 10–30 min interval of the test. This excellent resistance to plowing damage is attributed to the structural continuity arising from the isostructural CEIMs, as well as the excellent intrinsic hardness. Meanwhile, a high relative density of 94.13% greatly reduces stress concentration defects caused by pores, preventing severe wear from local high stress at the structural level and contributing to the stable friction behavior.

Based on the results of the tribological property tests and the surface morphology characteristics of the wear tracks, it can be determined that the dominant wear mechanisms of this composite material are early-stage abrasive wear, microcrack propagation, and localized delamination, with no adhesive wear and severe fatigue peeling occurring during the friction process. However, the excellent wear resistance can be attributed to the synergy of various mechanisms. Superhigh hardness: The fine grain structure increases the proportion of grain boundaries, and the CEIMs provide in-situ alternation of soft and hard micro regions without introducing a second phase. Different from the heterogeneous second-phase composite interface, the interface is separated by CEIMs monoboride solid solutions, which have the same boron skeleton and only occupy different metal sites, showing chemical gradient transition and crystallographic continuity. The continuity of the structure helps to mitigate interfacial debonding and delamination, and allows local composition modulation to passivate microcracks; When the relative density is 94.13%, the wear acceleration caused by stress concentration from the weak interface and holes can be weakened, but the contribution is limited. The combination of these structural and physicochemical mechanisms contributes to the low wear rate observed under the present testing conditions. It should be noted that due to the lack of a matched compositionally homogeneous reference sample, the individual contribution of the CEIMs to the mechanical and tribological properties cannot be quantitatively deconvoluted from other factors such as lattice distortion, grain size, and relative density. The observed performance synergy is therefore attributed to the combined effects of these high-entropy characteristics.

## 4. Conclusions

To develop new types of highly wear-resistant monoboride ceramics, we successfully prepared HEMBs composite ceramics (TiVCrMoW)B with stable structure and excellent performance by SPS. The inherent structure–property relationships among phase composition, microstructural characteristics, mechanical properties, and tribological behaviors of the material were systematically clarified. Specifically, the stabilizing effect of high configurational entropy enables the five components to form an orthogonal solid solution. Due to the influence of differences in atomic diffusion rates and chemical compatibility of elements, the material forms a special crystallographic matrix with homologous isostructural CEIMs that is macroscopically single-phase with selective segregation of elements such as Ti, W, and Mo. It spontaneously builds alternating distributed in-situ soft-hard isostructural microregions, which circumvents substantial interfacial mismatch and severe stress concentration induced by the formation of extrinsic heterogeneous second phases. The as-prepared (TiVCrMoW)B has a relative density as high as 94.13%. At a low load of 0.49 N, the Vickers hardness reaches 39.95 ± 4.32 GPa, and at 9.8 N, the Vickers hardness is 22.48 ± 1.26 GPa. Meanwhile, the fracture toughness is 3.39 ± 0.36 MPa·m^1/2^. More importantly, under a 10 N load, the volume wear rate of this material is as low as (1.11 ± 0.4) ×10^−6^ mm^3^/(N·m). Under comparable testing conditions, this value is close to that of high-entropy carbides and lower than that of high-entropy diborides, while noting the difference in test duration. The excellent tribological properties are the result of the synergistic effect of structural advantages and high-entropy effects. The homologous and isomorphous solid solution structure ensures the structural stability and toughness improvement of the composite material during the friction process. The inferred soft-hard microregions may help buffer the alternating stresses generated by reciprocating friction and absorb plastic strain energy; however, the local hardness contrast among these microregions was not directly measured and remains to be quantified by site-specific nanoindentation. However, there is no significant deterioration in hardness and toughness, resulting in only mild damage behavior such as slow expansion of microcracks, local layered peeling, and mild abrasive wear. The structural regulation strategy of homologous and isomorphous type proposed in this study avoids the interface defect problem of traditional multiphase modification, leading to concurrent improvement of hardness, toughness, and wear resistance under the present testing conditions. It provides a useful theoretical basis and technical reference for design, process optimization, and industrial application of refractory HEMB ceramics in extreme wear conditions.

## Figures and Tables

**Table 1:** Lattice parameters (a, b, c), measured bulk density (ρ), theoretical density (ρ_th_), and relative density of HEMBs-T and five compensate.

Compound	Space Group	Lattice Parameters			ρ (g/cm^3^)	ρ_th_ (g/cm^3^)	Ref.
		a (Å)	b (Å)	c (Å)			
HEMBs-T	Cmcm	3.12	8.23	3.00	7.86	8.35	This work
VB	Cmcm	3.04	8.04	2.97	/	5.65	[28]
CrB	Cmcm	2.97	7.86	2.93	/	6.10	[29]
MoB	Cmcm	3.16	8.51	3.09	/	8.53	[30]
WB	Cmcm	3.18	8.49	3.10	/	15.45	[31]
TiB	Cmcm	3.28	8.49	3.05	/	4.59	[32]

**Table 2:** Mechanical properties and wear resistance of HEMBs-T.

Composition	Vickers’ Hardness/GPa	COF	Volume Wear Rate/mm^3^/(N·m)	Fracture Toughness /MPa·m^1/2^	Flexural Strength/MPa
HEMBs-T	39.95 ± 4.32	0.52	(1.11 ± 0.40) × 10^−6^	3.39 ± 0.36 ^1^	243.3 ± 2.4

^1^ The fracture toughness is an apparent SENB value.

## Data Availability

The original contributions presented in this study are included in the article and supplementary materials. Further inquiries can be directed to the corresponding authors.

## Supplementary Materials

The following supporting information can be downloaded at: https://www.mdpi.com/article/10.3390/ma19214607/s1, Figure S1: characteristic peak of carbon element on sample surface detected by XPS Spectrum; Table S1: element content and proportion determined by EDS (%); Table S2: Surface roughness of specimens for friction and wear; Table S3: Estimated wear volumes based on four equally spaced cross-sectional profiles at four different locations in the representative wear test; Table S4: Wear volumes of the two additional independent friction and wear tests.
