# Supplementary material for: Synergistic Mechanical and Tribological Optimization of High-Entropy (TiVCrMoW)B Ceramics
Source: Materials (Basel). 2026 Oct 30;19(21):4607. doi: 10.3390/ma19214607 (PMC13764728; doi:10.3390/ma19214607)
Supplement: Supplementary file 1 [file materials-19-04607-s001.zip › materials-4632658-supplementary.pdf]

Supplementary Information

Synergistic Mechanical and Tribological Optimization of High-Entropy (TiVCrMoW)B Ceramics

Section S1

The fracture toughness (K_{IC}) is calculated by the single-edge notched beam (SENB) equation:

$$K_{IC} = \frac{PLF}{bh^{\frac{3}{2}}}$$

$$F = \frac{3}{2}\left(\frac{a}{h}\right)^{0.5} \times \frac{1.99 - \frac{a}{h}\left(1 - \frac{a}{h}\right) \times \left(2.15 - 3.93\frac{a}{h} + 2.7\left(\frac{a}{h}\right)^{2}\right)}{\left(1 + 2\frac{a}{h}\right)\left(1 - \frac{a}{h}\right)^{1.5}}$$

where P is the maximum load (in N), L is the support span (in mm), b is the specimen width (in mm), h is the specimen height (in mm), and a is the crack length (in mm).

Section S2

Table S1 element content and proportion determined by EDS (%)

**Spot**	**B**	**Ti**	**V**	**Cr**	**Mo**	**W**	**Total metal**	**metal ratio Ti:V:Cr:Mo:W**
**Spot** d	38.2	7.0	11.8	11.0	4.8	27.2	61.8	11.3:19.2:17.8:7.8:44.0
**Spot** e	59.5	3.6	1.9	5.5	27.5	2.0	40.5	8.8:4.7:13.7:67.9:4.9
**Spot** f	32.4	49.1	4.1	5.1	4.9	4.4	67.6	72.8:6.0:7.5:7.2:6.4
single-phase	50.0	10.0	10.0	10.0	10.0	10.0	50.0	10:10:10:10:10

Table S2 Surface roughness of specimens for friction and wear

Positions	Rz / µm
1	0.55
2	0.58
3	0.60

4	0.68
5	0.56
6	0.58
7	0.52
Average	0.58±0.05

Section S3

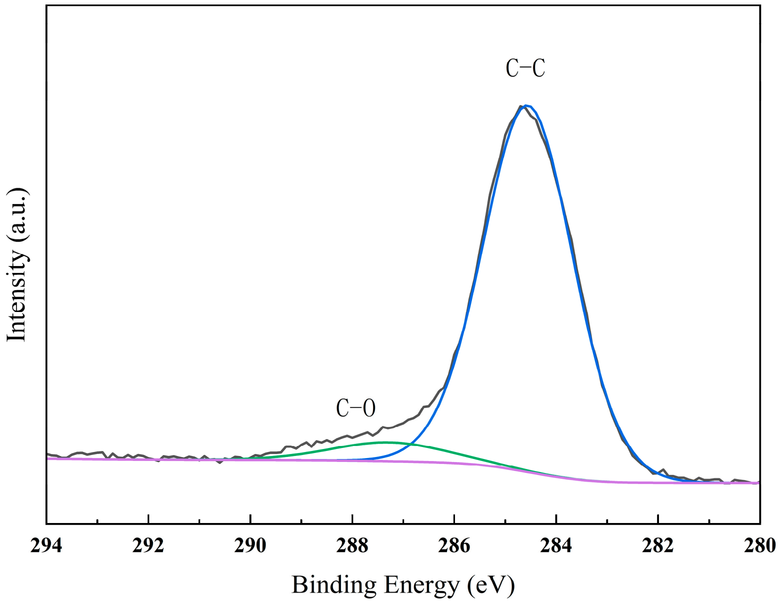

Figure S1 characteristic peak of carbon element on sample surface detected by XPS Spectrum

Section S4

For the representative test (Exp. 1), the four cross-sectional areas A_1-A_4 obtained through integration are 1.0×10^{-4} mm^2, 1.2×10^{-4} mm^2, 8×10^{-5} mm^2 and 1.0×10^{-4} mm^2, respectively. For each contour, the original reference plane $z_0(x)$ is determined by the un-abraded areas on both sides of the wear track. The cross-sectional area is calculated according to the following formula:

$$A_i = \int [z_0(x) - z(x)]\, dx$$

The wear volume $V_i = A_i \times l_{track}$. Here, l_{track} is the physical length of the wear mark, which is 5 mm. Through this process, the wear volumes obtained from the four profiles of the main wear test are listed in Table S3. For the two additional independent repeat tests (Exp. 2 and Exp. 3), six equally spaced cross-sectional profiles were collected for

each test, and the corresponding wear volumes are listed in Table S4.

Table S3 Estimated wear volumes based on four equally spaced cross-sectional profiles at four different locations in the representative wear test (Exp. 1)

Positions	Wear volume / mm^3
1	0.0005
2	0.0006
3	0.0004
4	0.0005
Average	$(5.0\pm0.8)\times10^{-4}$

Table S4 Wear volumes of the two additional independent friction and wear tests (Exp. 2 and Exp. 3). For each repeat test, six equally spaced cross-sectional positions (positions 1–6) were measured, giving 12 measurement positions in total.

Positions	Wear volume / mm^3	
	Exp. 2	Exp. 3
1	0.0009	0.0008
2	0.001	0.0008
3	0.0012	0.0009
4	0.0012	0.0008
5	0.0011	0.0008
6	0.001	0.0008
Average	$(1.07\pm0.12)\times10^{-3}$	$(8.17\pm0.41)\times10^{-4}$
